# Evans Blue Staining Reveals Vascular Leakage Associated with Focal Areas of Host-Parasite Interaction in Brains of Pigs Infected with *Taenia solium*

**DOI:** 10.1371/journal.pone.0097321

**Published:** 2014-06-10

**Authors:** Miguel Marzal, Cristina Guerra-Giraldez, Adriana Paredes, Carla Cangalaya, Andrea Rivera, Armando E. Gonzalez, Siddhartha Mahanty, Hector H. Garcia, Theodore E. Nash, The Cysticercosis Working Group in Peru

**Affiliations:** 1 Laboratory of Immunopathology in Neurocysticercosis, Facultad de Ciencias y Filosofía, Universidad Peruana Cayetano Heredia, Lima, Peru; 2 Facultad de Medicina Veterinaria, Universidad Nacional Mayor de San Marcos, Lima, Peru; 3 National Institutes of Allergy and Infections Diseases, National Institutes of Health, Bethesda, Maryland, United States of America; 4 Instituto Nacional de Ciencias Neurológicas, Lima, Peru; Biological Research Centre of the Hungarian Academy of Sciences, Hungary

## Abstract

Cysticidal drug treatment of viable *Taenia solium* brain parenchymal cysts leads to an acute pericystic host inflammatory response and blood brain barrier breakdown (BBB), commonly resulting in seizures. Naturally infected pigs, untreated or treated one time with praziquantel were sacrificed at 48 hr and 120 hr following the injection of Evans blue (EB) to assess the effect of treatment on larval parasites and surrounding tissue. Examination of harvested non encapsulated muscle cysts unexpectedly revealed one or more small, focal round region(s) of Evans blue dye infiltration (REBI) on the surface of otherwise non dye-stained muscle cysts. Histopathological analysis of REBI revealed focal areas of eosinophil-rich inflammatory infiltrates that migrated from the capsule into the tegument and internal structures of the parasite. In addition some encapsulated brain cysts, in which the presence of REBI could not be directly assessed, showed histopathology identical to that of the REBI. Muscle cysts with REBI were more frequent in pigs that had received praziquantel (6.6% of 3736 cysts; n = 6 pigs) than in those that were untreated (0.2% of 3172 cysts; n = 2 pigs). Similar results were found in the brain, where 20.7% of 29 cysts showed histopathology identical to muscle REBI cysts in praziquantel-treated pigs compared to the 4.3% of 47 cysts in untreated pigs. Closer examination of REBI infiltrates showed that EB was taken up only by eosinophils, a major component of the cellular infiltrates, which likely explains persistence of EB in the REBI. REBI likely represent early damaging host responses to *T. solium* cysts and highlight the focal nature of this initial host response and the importance of eosinophils at sites of host-parasite interaction. These findings suggest new avenues for immunomodulation to reduce inflammatory side effects of anthelmintic therapy.

## Introduction

Neurocysticercosis (NCC) is an infection of the central nervous system with metacestodes (cysts) of the cestode *Taenia solium*. Although cysts can be found anywhere in the body, they develop mostly in the muscles, subcutaneous tissues, and brain. Almost all the symptoms in humans, most commonly seizures, are the consequence of involvement of the brain [1], [2].

Viable cysts in the brain show little or no pericystic inflammation. However, the host begins to react to the degenerating cyst during the natural course of the infection or as a result of anthelmintic treatment, and an intense inflammation commonly ensues [1]. Over time, the cyst undergoes a series of degenerative changes resulting in granuloma formation, consolidation and, frequently, calcification [3], [4]. Treatment induced inflammation is a major concern in disease management since it results in higher frequency of seizures soon after treatment and, therefore, complicates treatment [2], [5]. The nature of the induced inflammation has not been studied in humans but a few studies have been previously performed in pigs [6]–[8]. Notably, how to best control acute treatment induced inflammation has not been systematically studied.

The pig is the natural intermediate host of *T. solium* and infection results in cysts that commonly develop in the brain and muscle, similar to human infections. Pigs and humans have similar histopathological changes in brain and muscle, indicative of parasite damage and inflammatory response surrounding the cyst [9], [10]. Therefore, infected pigs can serve as a good model for the study of human NCC. Although infected pigs have been studied previously [11]–[15], they have not been used to systematically characterize treatment-induced inflammation and pathology.

Pericystic inflammation is characteristic of the host response to degenerating cysts in *T. solium* infected pigs and cysts of other species of cestodes used in animal model infections [6]–[8], [16]–[19]. In the brain, inflammation around cysts induces breakdown of the BBB, a loss of cerebrovascular integrity, which can be identified with a variety of tracers [20]–[23] including intravenously injected Evans blue (EB). Extravasation of EB, a vital dye that binds predominantly to albumin in vivo [24], is one technique that has been used extensively to study disruption of the BBB in a variety of settings, including ischemic injury [25], [26] and infections of cerebrospinal spaces [27], [28]. We developed an acute treatment model and employed EB to detect increased permeability of the BBB [29]. Using this model we performed a number of immunological and histological based analyses on blue dyed and undyed encapsulated brain cysts over time following treatment, which are the subject of another report. Here we describe and characterize the presence of one or more focal EB infiltrated regions (REBI) on the surface of non-encapsulated muscle *T. solium* cysts, their histological equivalents in encapsulated brain cysts, their association with praziquantel treatment and the why REBI are dyed with EB.

## Methods

### Study population and specimen collection

Detection and analysis of cysts containing REBI was performed as part of a study described earlier of the usefulness of injecting the dye to delineate BBB dysfunction in treated and untreated pigs. The methods employed for treatment, EB injection, treatment and collection of samples have been reported [29]. Briefly, eight heavily infected pigs were treated with a single oral dose of praziquantel (100 mg/kg; 10% Saniquantel, Montana S.A, Peru); four were euthanized at 48 hr (PZQ48) and four were euthanized 120 hr (PZQ120) post treatment. Three additional infected pigs did not receive the anthelmintic and served as untreated controls. Two hours before sacrifice, all pigs were anesthetized and injected via the carotid artery with 80 mg/kg EB (Sigma-Aldrich, St. Louis, MO) using a 2% solution of EB in saline solution (0.85% sodium chloride; Laboratorios Baxter, Peru). Immediately after sacrifice, the pigs were perfused with cold saline solution containing sodium heparin (10 IU/mL; L&S Medical S.A., Peru). Finally, non encapsulated muscle cysts and brain and muscles tissues containing encapsulated cysts were processed for histological evaluation. EB infusion and necropsies of all eleven animals were performed on six different dates [29]. The maintenance and care of experimental animals and all procedures described comply with the Animal Ethics and Wellbeing Committee of the Facultad de Medicina Veterinaria, Universidad Nacional Mayor de San Marcos, Lima, Peru.

### Processing of specimens

Three subsets of specimens were collected shortly after necropsy: non-encapsulated muscle cysts, encapsulated muscle cysts and encapsulated brain cysts; the term capsule refers to pericystic tissue composed by host inflammatory cells, collagen and blood vessels. All specimens destined for histopathological studies were immediately fixed in 10% neutral buffered formalin (37% formaldehyde in phosphate buffered solution, pH 7.2) for 24–30 hr and paraffin-embedded for sectioning. Serial sections (4 µm in thickness) were mounted on poly-L-Lysine-coated slides (Sigma, St. Louis, MO) and stained with hematoxylin and eosin (H&E) as well as Masson's trichrome stain for histological analysis. Slides were examined with a light microscope (Primo Star, Zeiss, Germany) and photographed with a calibrated camera (AxioCam ICc1, Zeiss, Germany) using AxioVision software (version 4.6, Zeiss, Germany). Encapsulated muscle and brain cysts were classified by gross examination as clear or EB dyed.

### Histological characterization of REBI in non encapsulated muscle cysts

Some non encapsulated muscle cysts, excised after EB injection and necropsy, exhibited one or more clearly visible small, round foci of superficial EB infiltrated regions (REBI). These cysts, devoid of the capsular host tissue, were selected and processed as above to define the histological characteristics of the focally stained areas.

### Histological characterization of REBI in encapsulated muscle and brain cysts

Since gross identification of a cyst with REBI is not possible when the cyst surface is obscured from view by a capsule, a histological approach was taken to determine the capsular inflammation associated with these regions. For this, 10 muscle cysts in paraffin blocks with diffuse EB stained capsules and a limited region in the cyst wall suggestive of REBI were examined histologically to determine the presence of REBI and the capsular inflammation associated with these regions. As a rule, 50 serial sections, which included the entire encapsulated cyst, were examined; three cysts were identified that showed histopathology identical to REBI. To identify brain cysts with identical histopathogy to REBI, every encapsulated brain cyst was serially sectioned.

### Histological characterization of the inflammatory pathology

An adaptation of a previously reported scheme for grading cellular infiltrates and cyst wall damage was used to characterize the extent of the inflammatory infiltrates and cyst destruction associated with REBI [9], [10]. Inflammation was described as mild, moderate, strong or intense, depending on the types and abundance of inflammatory cells. The degree of cyst wall damage for each cyst, with or without capsule, was also described as mild, moderate, strong or intense, from 1 to 4 as described earlier [9], [10] depending on the integrity of the histological layers.

### Identification of inflammatory cells containing EB in REBI

The cells responsible for EB uptake that constituted REBI were investigated taking advantage of the light EB presence in REBI in deparaffinized, unstained sections, as observed by bright field microscopy, and red autofluorescence of EB [30], [31] observed on the same slides using excitation and emission filters for rhodamine fluorescence (Zeiss D-7082 inverted fluorescence microscope, Zeiss, Oberkochen, Germany). These slides were then stained with hematoxylin and eosin (H&E) to assess cell histopathology. Sections contiguous to those stained with H&E were analyzed with intracellular eosinophil peroxidase (EPX). EPX was localized with a mouse anti-mouse EPX antibody (mEPX), diluted at 1∶500 (a gift from Dr. J.J. Lee at the Mayo Clinic, Scottsdale, Az). All immunostaining was performed on a Leica Bond Max autostainer (Leica Microsystems, Bannockburn, IL) using the BondMax biotin-avidin free polymer-based detection system preceeded by heat-induced epitope retrieval with Leica retrieval solution (citrate buffer, pH 6.0) for 25 min. 3,3′-Diaminobenzidine was used as the chromogen. Sections were examined and photographed on a Leica epifluorescence microscope (Leica Microsystems, Bannockburn, IL).

### Statistical analysis

The frequencies of cysts with REBI were compared between groups with the Fisher's exact and Chi-square tests. Differences with p-values≤0.05 were considered statistically significant. Statistical analyses were performed using Stata 11 (Statacorp, College Station, Texas).

### Ethical statement

Animal experiments were performed at the Facultad de Medicina Veterinaria, Universidad Nacional Mayor de San Marcos, Lima, Peru, under the protocol “Evaluación de la permeabilidad vascular en cerebro y músculo de cerdos naturalmente infectados con *Taenia solium*”, with Dr. Armando Gonzalez as principal investigator. The study protocol was approved by the Animal Ethics and Wellbeing Committee of the University. The Laboratory Animal Care and Use Guidelines on Training is registered with the National Institutes of Health and follows their guidelines. In addition it follows the guidelines of the Royal Society for the Prevention and Cruelty to Animals, the British Veterinary Association, the Fund for Replacement of Animals in Medical Experiments, the Universities Federation for Animal Welfare and Guidelines for the Care and Use of Mammals in Neuroscience and Behavioral Research. The study was registered with both Universidad Peruana Cayetano Heredia and Universidad Nacional Mayor de San Marcos.

## Results

**Figure 1:**
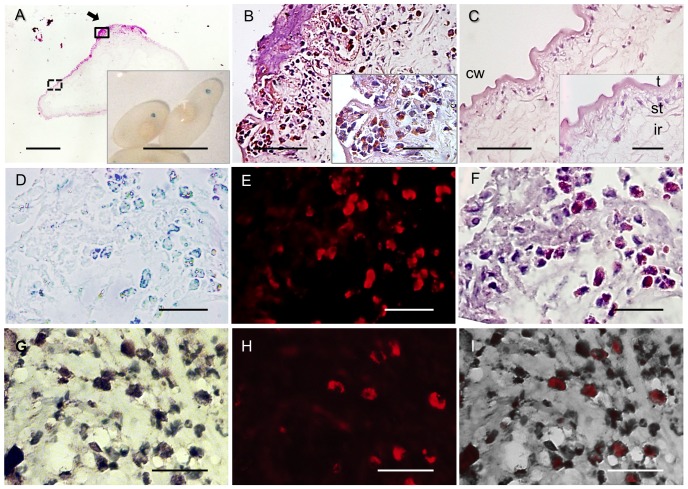
Characterization of REBI in muscle cysts. (**A**) A representative hematoxylin-eosin (H&E) stained section through a REBI (black arrow) on a non encapsulated muscle cyst; the solid rectangle represents the area shown in “B”; isolated, non encapsulated, muscle cysts demonstrating REBI (inset). (**B**) A high and even higher magnification view (inset) of the eosinophil-rich cellular infiltrate of the REBI shown in “A” (solid rectangle). (**C**) Uninvolved cyst wall showing identifiable structures in a region close to the REBI (inset) in two magnified views of the dashed line rectangle in panel “A”. (**D**) Bright field microscopy of an unstained deparaffinized section of the REBI of the muscle cyst in “A”, demonstrating uptake of EB by eosinophils. (**E**) Fluorescent microscopy with a rhodamine filter of the same section. (**F**) H&E staining of the same section. (**G**) Immunolocalization of eosinophils and EB in a REBI with eosinophil peroxidase (EPX)-specific antibodies (brown interior staining). (**H**) Fluorescence micrograph of the same section as shown in “G”. (**I**) Merged image of “G” and “H”. Bar: 1 µm (A inset); 400 µm (A); 200 µm (B, C); 20 µm (D — I and insets in B and C). Key: cw = cyst wall; t = tegument; st = subtegument; ir = internal region.

While investigating the association between disruption of the BBB and inflammation due to infection by *T. solium* and anthelmintic treatment in pigs [29], the presence of one or more small, round foci of Evans blue infiltration (REBI) was incidentally noted on the surface of non encapsulated cysts extracted from host muscle tissue (Fig. 1A, inset). REBI was easily distinguishable from diffusely EB dyed cysts, in which extensive or global EB dyeing was visible throughout the host-derived capsule. REBI areas were present anywhere on the surface and were not limited to a particular anatomic region of the cyst.

### Anthelmintic treatment was associated with a higher frequency of non encapsulated muscles cysts with REBI

The proportion of REBI in muscle cysts was determined by inspecting all the harvested cysts from only the right hind leg. REBI containing cysts were detected in the muscles in 6 of 8 infected pigs assessed; in three pigs muscle cysts were not evaluated. Six of 3172 cysts (0.2%) from untreated pigs had REBI, whereas the corresponding frequencies of REBI in cysts from PZQ-treated pigs were 126 of 2750 cysts (4.6%) from the PZQ48 group and 119 of 986 cysts (12.1%) in the PZQ120 group, both of which were significantly higher (p<0.001; Fisher's Exact test). Among the treated groups (those studied at 48 h and 120 h post-treatment), the proportion of cysts was higher at 120 hr compared to 48 hr (12.1% vs. 4.6%, respectively; p<0.001) (Table 1). These data demonstrate a direct association between anthelmintic treatment and the presence of REBI.

**Table 1:** Frequencies of non-encapsulated muscle cysts with REBI from untreated and PZQ-treated pigs.

	Pigs	Total cysts	Cysts		% Cysts with REBI	Fisher's exact test
			without REBI	with REBI		
UT	2	3172	3166	6	0.2	–
PZQ48	3	2750	2624	126	4.6	p<0.001*
PZQ120	3	986	867	119	12.1	p<0.001*
						p<0.001**
All PZQ	6	3736	3491	245	6.6	p<0.001*
Total	8	6908	6657	251	3.6	

* compared to UT.

** compared to PZQ48.

### EB was localized in eosinophils present within REBI in non-encapsulated muscle cysts

Non-encapsulated muscle cysts with REBI were sectioned through the involved regions for histological examination to characterize the pathological changes associated with the BBB disruption represented by the REBI. H&E staining revealed that REBI co-localized to focal regions of inflammatory infiltrate of varied intensities that penetrated the cyst wall (Fig. 1A, 1B). Cellular infiltrates were associated with different degrees of damage to the cyst wall layers. Notably, outside the REBI areas, there was little or no inflammatory infiltrate and the layers of the cyst wall were undamaged (Fig. 1C). The focal inflammatory infiltrate was characterized by a predominance of eosinophils, moderate numbers of macrophages and lymphocytes, and occasional plasma cells, the density of which increased with the severity of the inflammation and the level of damage to the cyst wall structures. In some cysts all layers of the cyst wall were altered or damaged, with the outermost layer (tegument) demonstrating the most severe damage. In such cases, abundant normal and degranulated eosinophils and other inflammatory cells and degenerating parasite material formed a dense amorphous mass inside the cyst wall. Some muscle cysts possessed multiple REBI areas, all of which were histopathologically indistinguishable from single REBI.

Serial sections of deparaffinized, unstained REBI in non encapsulated muscle cysts were examined under light and fluorescence microscopy to identify and co-localize EB with the cells involved in EB uptake. A subpopulation of inflammatory cells in REBI demonstrated a light blue cytoplasm (Fig. 1D) and exhibited a brilliant red cytoplasmic fluorescence in the rhodamine fluorescence spectrum (Fig. 1E), characteristic of EB autofluorescence [30], [31]. Microscopic examination of these sections following with H&E staining identified the EB containing cells as eosinophils (Fig. 1F). Importantly, eosinophils containing EB were present in all REBI cysts analyzed. To confirm that eosinophils had internalized EB, mouse anti-eosinophil peroxidase (EPX) antibody co-localized to cells with the red fluorescence of EB (Fig. 1G, 1H, 1I). However, only a subset of eosinophils internalized EB. No other cell type showed EB dyeing.

### The same histopathology as in REBI was identified in encapsulated cysts from muscle and brain encapsulated cysts

**Figure 2:**
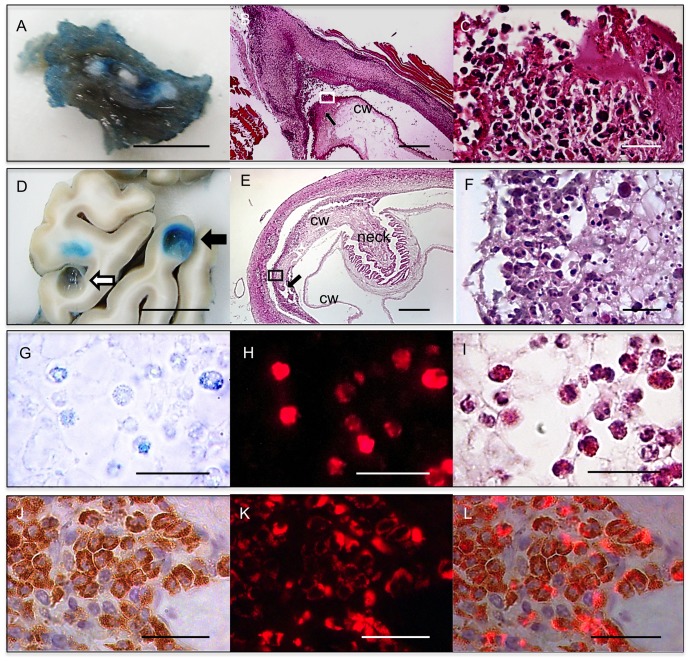
Histopathology of putative REBI on encapsulated muscle and brain cysts. (**A**) Gross view of muscle tissue with encapsulated cysts. (**B**, **E**) H&E-stained sections of cysts showing the locations of putative REBI regions (black arrow) in encapsulated muscle (**B**) and brain (**E**) cysts. (**C**, **F**) Magnified views of the putative REBI region on encapsulated muscle (**C**) and brain (**F**) cysts stained with H&E, squares in “B” and “E” indicate the portions of the cyst wall (cw) that are magnified in “C” and “F”. (**D**) Part of a brain coronal section showing a clear (white arrow) and an encapsulated cyst (black arrow) with a REBI capsule. (**G**) Bright field microscopy of a deparaffinized but non-stained section through a putative REBI of a brain cyst showing uptake of EB by eosinophils. (**H**) Fluorescence microscopy, as described in Fig. 1, on the same section shown in “G”. (**I**) H&E staining of the section adjacent to “H”. (**J**) Immunolocalization of EPX on a putative REBI (brown interior staining). (**K**) The same section viewed with a rhodamine filter. (**L**) Merged image of J and K. Bar: 1 cm (A, D); 400 µm (B, E); 20 µm (C, F, G — L).

REBI were evident in non-encapsulated muscle cysts only because their capsules had been stripped away in the process of collecting cysts. Therefore, the histopathology of the capsule contiguous to REBI could not be directly evaluated. However, systematic sequential sectioning (see Methods) and histological analysis of some encapsulated muscle cysts with diffuse EB staining (blue cysts) (Fig. 2A) showed histopathology that was identical to that seen in cysts with REBI. Hence, these can be assumed to be encapsulated cysts with REBI histopathology (Fig. 2B, 2C).

All 151 encapsulated brain cysts were processed and analyzed (Fig. 2D). None of the 29 clear brain cysts showed REBI histopathology. In contrast 8 of 122 encapsulated cysts with diffuse EB dyeing (2 from the PZQ48 group and 6 from the PZQ120 group) demonstrated REBI histopathology. (Fig. 2E, 2F).

More encapsulated cysts with REBI histopathology were found in the brains of the PZQ120 group than in the UT (p<0.005) and PZQ48 group (p<0.01). The number of cysts with REBI histopathology was not statistically different in the PZQ48 group compared to the number in the untreated group, but the number of cysts with REBI histopathology was significantly higher in all treated than in untreated pigs (p<0.05; Table 2).

**Table 2:** Frequencies of encapsulated brain cysts with putative REBI from untreated and PZQ-treated pigs.

	Pigs	Total cysts	Cysts		% Cysts with REBI	Fisher's exact test
			Without REBI	With REBI		
UT	3	47	45	2	4.3	–
PZQ48	4	75	73	2	2.7	NS*
PZQ120	4	29	23	6	20.7	p<0.005*
						p<0.01**
All PZQ	8	104	96	8	7.7	p<0.05*
Total	11	151	143	10	6.6	

* compared to UT.

** compared to PZQ48.

NS: non significant.

The capsules of the 8 brain cysts with REBI histopathology showed focal regions of very intense inflammation extending into the wall and body of the cyst at the inflammatory site, which was the only part of the parasite affected (Fig. 2E). Contiguous with the inflammatory infiltrate at REBI regions, the cyst walls had levels of damage ranging from mild to severe. Similar damage was present in encapsulated muscle cysts with REBI histopathology. The focal regions of inflammation penetrating the cyst wall were composed of intense eosinophilic infiltrates intermingled with mononuclear cells and amorphous debris.

Serial sections of both brain and muscle encapsulated cysts with REBI histopathology confirmed that the cyst wall outside the involved regions was normal, lacked inflammatory cells and was undamaged, while the surrounding capsules demonstrated varying degrees of inflammation.

**Figure 3:**
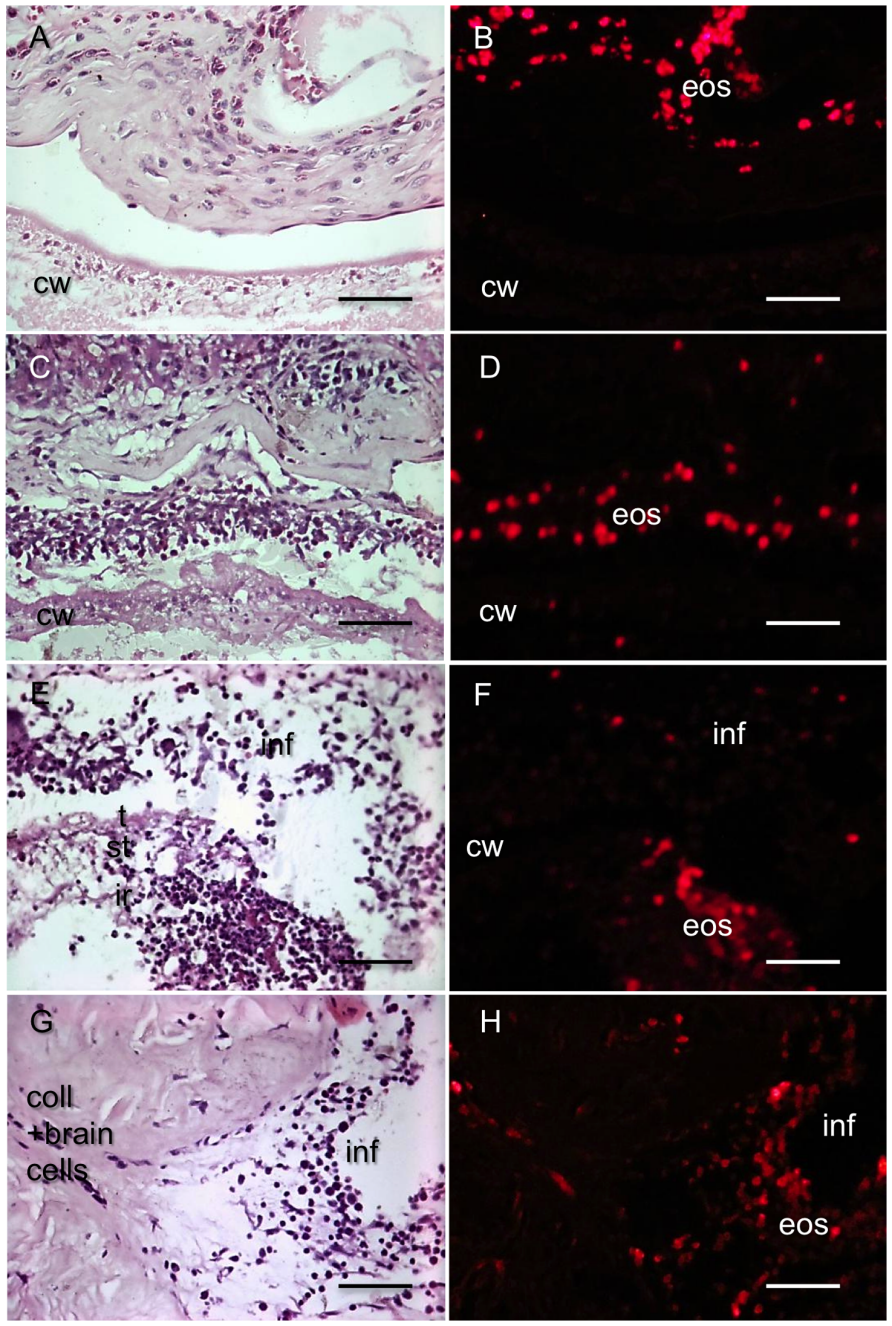
Distribution of EB uptake in eosinophils in host infiltrates associated with REBI and brain inflammation of differing severity. H&E staining (**A**, **C**, **E**, **G**) and fluorescence microscopy (**B**, **D**, **F**, **H**) of brain sections from infected pigs with encapsulated cysts using a rhodamine filter. Inflammatory infiltrates and adjacent cyst walls showing moderate inflammation (**A** and **B**), strong inflammation (**C** and **D**); a REBI (**E** and **F**) and severe inflammation (**G** and **H**). Key: cw = cyst wall; inf = infiltrate; t = tegument; st = subtegument; ir = internal region, col = collagen fibers. Bar: 20 µm.

The identification of the EB-containing cells in encapsulated brain cysts with REBI histopathology was performed in the same way as for REBI characterized in non- encapsulated muscle cysts. Similar to the muscle cysts with REBI, encapsulated brain cysts with REBI histopathology also had blue dyeing in a proportion of the cells indicating uptake of EB (Fig. 2G). Further investigation confirmed co-localization of EB autofluorescence in cells with the morphology of eosinophils and with mEPX antibody reactivity. These results confirm that a subset of eosinophils had ingested EB (Fig. 2H–L, Fig. 3), further strengthening the evidence that these are indeed encapsulated REBI cysts.

Eosinophils that had taken up EB were present in capsules with a broad range of inflammatory responses but, in general, the proportion of eosinophils increased as the inflammation score went from moderate (Fig. 3A, 3B) to intense (Fig. 3C, 3D); in the strongest and most advanced stage of inflammation, eosinophils had penetrated the wall into the interior structures of the cysts (Fig. 3G, 3H).

## Discussion

The major findings of this work are recognition, description and characterization of focal regions of Evans blue infiltration (REBI) on the surface of *T. solium* cysts, which we found are circumscribed regions of BBB disruption associated with inflammatory responses. Furthermore, regions with identical histopathology were identified in encapsulated muscle and brain cysts.

Although previous studies examined untreated and treated pigs and described focal eosinophilic-rich inflammation directed to cysts [6]–[8], there are major differences between earlier descriptions and REBI. This is not surprising since REBI is defined functionally as the presence of EB dyed structure on the cyst wall and then characterized histopathologically. In contrast, prior studies defined histopathological microscopically defined stages of inflammation and cyst damage of increasing involvement and severity at one time point based upon autopsy findings of randomly selected untreated pigs. In this study the first sign of a host response was seen opposite the spiral groove of the cyst. In contrast, REBI described in the current study are macroscopic regions of host attack that were present at many locations on the cyst, not limited to a specific anatomic region of the cyst. Additionally we were able to assign a likely early time of formation of REBI, since we documented increased proportion of affected cysts 48 and 120 hr following a single dose of praziquantel. Therefore the formation of REBI is an early inflammatory response to the cyst, although not necessarily the earliest since it is assumed capsular inflammatory responses precede cyst wall located inflammation. Although there is much in common between REBI and prior descriptions of the histopathology of the host response to cysts in pigs, what was not observed before is one or more distinct functional small focal regions of inflammatory attack directed to the cyst that is best appreciated shortly after praziquantel treatment. We did not see initial responses limited to the region of the spiral canal, as described in earlier studies and mentioned as focal local areas of inflammatory response.

As noted above, REBI are sites of an early effective, damaging host response to the cyst. We can base this conclusion on a number of features of the REBI. Firstly, the inflammation associated with the REBI is limited to small focal, circumscribed region(s) on the tegument of affected cysts and contrasts with the general inflammatory response seen in the majority of degenerating cysts. Secondly, the frequency of cysts with REBI increases following praziquantel treatment, which triggers inflammatory responses to viable cysts. Thirdly, the timing of the appearance of REBI at 5 days is similar to the time frame in which humans develop symptoms/seizures following initiation of treatment, generally occurring within the first week post-treatment. The number of REBI cysts is a minority of the total number of cysts; one reason for the low frequency may be because the cysts were sampled at one point in time in the course of an evolving process, which may not be the optimal time for the development of REBI in all cysts. Another potential reason for the low frequency of REBI could be the relatively low efficacy of a single dose of praziquantel – only a proportion of cysts may have been damaged of killed. Whether REBI represent the only initial or early response or a special type of response can only be ascertained through prospective analysis of cysts in treated animals over an extended period of time.

REBI can only be directly visualized on cysts devoid of their capsules; our initial discovery of REBI containing cysts occurred in the process of harvesting muscle cysts extruded from their capsules for other experiments [29]. Because capsules are the primary host response to the cyst and approximate the cyst wall, we searched in cysts with intact capsules (such as those in the brain) for evidence of cyst inflammation identical to that found in REBI adjacent to areas of capsular inflammation. This allowed direct visualization of extension of the capsular inflammation into the cyst wall and also to estimate the proportion of encapsulated brain cysts with REBI. Using this definition, we found that the proportion of encapsulated brain cysts with REBI increased progressively with time after praziquantel treatment, similar to muscle cysts. Also, all cysts with REBI demonstrated EB dyed capsules, which indicates an extensive capsular vascular BBB dysfunction and inflammatory response within the capsule. This logically leads us to postulate capsular inflammation precedes the presence of REBI since inflammatory cells can only originate from outside the cyst.

The inflammatory response in swine with NCC has been described by a number of investigators [6], [8], [32], [33]. The overall inflammatory response to cysts is not further characterized here except as it pertains to the description of the cellular response present in REBI. The latter consisted primarily of mononuclear cells, lymphocytes and eosinophils, which became more numerous as the intensity and severity of the inflammatory response increased. The inflammation in the REBI is in many ways typical of the description of the inflammatory reactions to cysts in swine [9], [10]. Our impression is that proportion and number of of eosinophils appears to be consistently high or higher in REBI than in usual host inflammation to cysts. Further characterization of the cellular infiltrate of REBI was not performed and would have been informative. However, use of immunohistopathological characterization of cellular infiltrates in pigs is significantly constrained because of a general lack of usable antibodies, which are commonly either uncharacterized, non reactive, not specific or not available. So although immunohistopathology is greatly rewarding in other common laboratory mammalian hosts such as mice and in humans, it is limited in the pig. For this reason, we have not further characterized the REBI infiltrates using this methology and instead rely upon RT PCR analysis in our more extensive studies (unpublished results).

Eosinophils predominate in these inflammatory infiltrates (Fig. 1I, 2I), at times forming a layer of cells at the cyst capsular interface [6]. A surprising finding was the presence of intracellularly located EB in some but not all eosinophils, which was confirmed using two different histopathological methodologies. Since EB is bound tightly to albumin [24] this indicates that albumin is taken up by eosinophils. The reason uptake occurred preferentially or selectively in eosinophils is unknown. As mentioned, EB uptake could not be evaluated dynamically, and it is possible that other phagocytic cell populations (such as macrophages [34]) take up EB at other time points, with different kinetics. Exposure of human albumin to eosinophils in vitro stimulates migration via PI3 kinase activation and likely induces an activated phenotype with an increase in size [35]. Whether this induces phagocytosis of albumin is unknown. Eosinophils infiltrating into *T. solium* capsules may also be activated, leading to albumin ingestion along with EB. Other molecules that are carried by albumin may also be important in facilitating uptake of EB. Why some eosinophils take up EB and some do not is unclear but may have to do with factors such as differing activation states of eosinophils in REBI at the time of EB injection.

Since our methods are not sufficiently quantitative to determine the amount of EB present in tissues, we cannot determine the contribution of eosinophil uptake to total EB staining. However, a notable observation was that we did not find other cells containing EB, nor did we see extracellular EB or EB near vessels, which might be expected after disruption of the vascular barrier. Also, we found no evidence that the wall of the cyst itself was dyed with EB. Thus, the uptake of EB was predominantly, if not exclusively, by eosinophils.

Eosinophils are known to play an essential role in the cytotoxic response to helminth parasites and were identified in the inflammatory infiltrate around viable *T. solium* cysts in pig muscles [33]. Eosinophils have a direct role for in the killing of many multicellular pathogens such as *Schistosoma mansoni* and *S. haematobium*, [36], possibly through proteolytic enzymes such as eosinophil granular proteins released by degranulating eosinophils [37], antibody-mediated cytotoxicity [36], [38]–[40] and activation of phagocytes through release of cytokines and chemokines [41]–[43]. The presence of eosinophils among the inflammatory cell infiltrates has been previously reported [6], [32] and seems to be a common feature of the inflammation.

In humans, cysticidal treatment incites an intense inflammation as noted clinically by seizures and the new appearance of pericystic edema and enhancement [4], [44], which are the imaging correlates of inflammation. Indeed, this post-treatment inflammation is a major issue in the management of anthelmintic therapy in patients with live cysts. These data point to a potential role of eosinophils in the induction of treatment-related side effects and suggest directed strategies that could be developed to inhibit this inflammatory reaction. The porcine model described provides a useful tool to elucidate the mechanisms controlling pericystic inflammation and will therefore assist in developing therapeutic strategies to suppress the pathological inflammation that is characteristic of this complex and debilitating disease.
